# An overview of the rare parotid gland cancer

**DOI:** 10.1186/1758-3284-3-40

**Published:** 2011-09-14

**Authors:** Kimberley Ho, Helen Lin, David K Ann, Peiguo G Chu, Yun Yen

**Affiliations:** 1 Department of Molecular Pharmacology, Beckman Research Institute, City of Hope National Medical Center, Duarte, CA 91010, USA; 2 Department of Pathology, City of Hope National Medical Center, Duarte, CA 91010, USA

**Keywords:** Parotid gland cancer, salivary gland cancer, targeted therapeutics, vacuolar protein sorting-associated protein 4B, vascular endothelial growth factor, epidermal growth factor receptor, endosomal sorting complexes required for transport, multi-vesicular bodies

## Abstract

Cancer of the parotid gland is relatively rare, but carries poor prognosis owing to its prevailing distant metastases. In addition to the disease's basic epidemiology and pathology, we review some current discoveries of its tumorigenesis molecular mechanism. Based on published salivary gland cancer clinical trial data, non-surgical antitumor efficacies amongst a range of chemotherapy, radiation, and concurrent therapy regimens are compared. We also present the current development status of novel radiation therapy and targeted therapeutics, focusing on intensity-modulated radiation therapy (IMRT), and epidermal growth factor receptor (EGFR) and vascular endothelial growth factor (VEGF) blockages, which are showing promise for improving parotid gland cancer management.

## Anatomy

**Figure 1:**
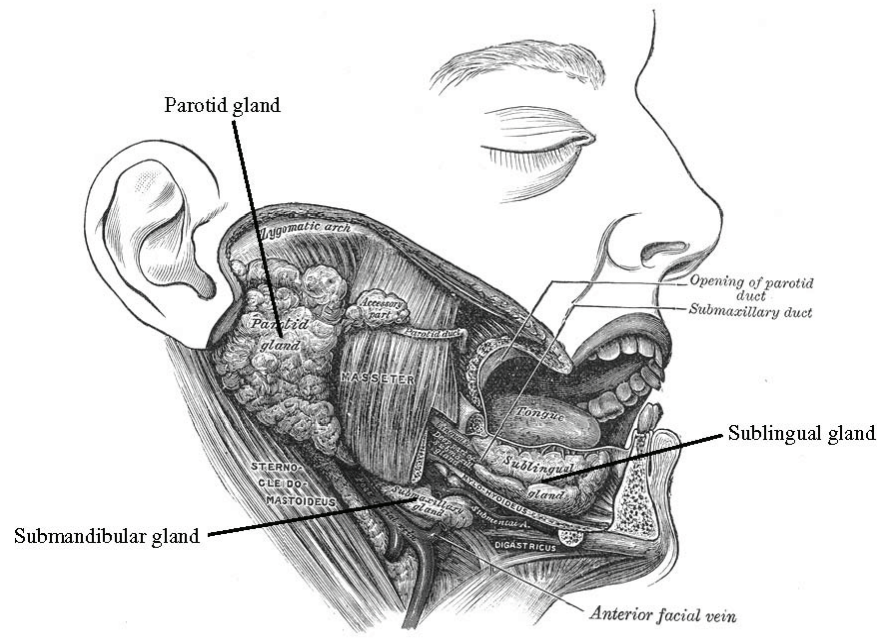
**Ilustration of a facial dissection with major salivary glands identified **[1].

The salivary glands are important organs in organisms since they serve as exocrine glands in the secretion of saliva and the enzyme amylase into the oral cavity to facilitate mastication and swallowing. There are three pairs of major salivary glands: the sublingual glands that are located beneath the tongue, the submandibular glands that are located below the lower jaw, and the parotid glands that are located in front of the ears and extend to the area beneath the earlobe along the lower border of the jawbone. The parotid glands are the body's largest salivary glands (See Figure 1) [1]. The minor salivary glands are small glands that number in the hundreds are located in the lips, bucked mucosa, and throat linings.

## Epidemiology

**Figure 2:**
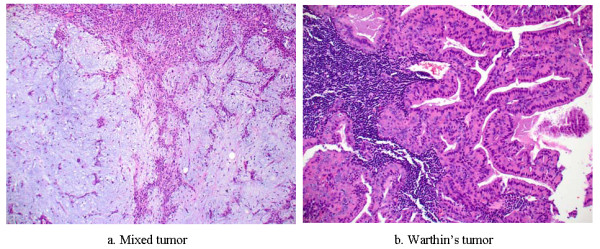
**Salivary gland benign tumor pathology (200 ×)**.

Compared to other cancers, salivary gland malignancies are relatively rare in the United States. In 2008, they comprised only about 12% of oral & pharyngeal cancers or 0 · 3% of cancers at all sites combined [2]. More cases have also been noticed in areas of higher ultraviolet radiation [3]. During the 2000-2008 period, salivary gland malignancies occurred more often in men at an average annual incidence rate of 1 · 41 cases per 100,000 males than in women at 1 · 00 [4]. Through the past few years, its incidence has also slowly increased among men at about 1 · 2% per year [2]. Although it is possible for salivary gland cancer to occur in people of all ages, 2 out of 3 cancers are found in people 55 and older. On average, people are diagnosed at age of 64 [5]. Most salivary gland tumors are benign. The most common benign tumors are mixed tumor and Warthin's tumor (See Figure 2a-b).

Salivary gland tumors account for about 5% of all neoplasms of the head and neck. Most (75%) occur in the parotid glands, which are the largest among the three sets of major salivary glands, 10% arise in the submandibular glands, and 15% are located in minor salivary glands of the upper digestive tract, less than 1% present in the sublingual glands [6]. Only about 20% of parotid gland tumors are malignant. Half of submandibular and sublingual tumors, and 20% of the minor salivary gland tumors are benign [7]. The five-year relative survival rate for salivary gland cancer depends on the stage the cancer. From Stage I to IV, the rates are 96%, 77%, 73%, and 37%, respectively [8].

## Etiology

Partly owning to the rarity of parotid gland cancer, its etiology has not been thoroughly studied and the factors responsible for its carcinogenesis are unclear. Exposures to tobacco smoke and alcohol intake have not been found consistently associated with its development. However, one of the well-established risk factors is exposure to ionizing radiation, as supported by studies on atomic bomb survivors [9]. Linear dose-dependent relationships have also been observed [10]. Medical radiation or ultraviolet light therapeutic treatments to the head or neck and exposures to full-mouth dental X-rays have also been linked to an increased risk [11]. The effect of UV therapeutic light seems to be more evident in fair-skinned persons, who are naturally more sensitive to the effects of UV light [12]. Additionally, nitroso compounds have induced parotid gland tumors in laboratory mice. The presence of nitroso compounds in rubbers could explain the higher incidence of parotid gland cancer in rubber industrial workers [13].

## Classification

Mucoepidermoid carcinoma is most common cancer in the parotid gland. Approximately 50% of submandibular gland cancers are adenoid cystic carcinomas. Minor salivary gland cancers are most often adenoid cystic carcinomas and adenocarcinomas, not otherwise classified [14]. Table 1 shows the histologic types of salivary gland cancer in order of frequency [15].

**Table 1:** Frequency of salivary gland cancer by histologic type

Histologic Type	Frequency of Occurrence	Distribution
Mucoepidermoid carcinoma	34%	Most common malignant parotid gland tumor, 40-50% of cases
Adenoid cystic carcinoma	22%	Most frequent in palate and submaxillary gland
Adenocarcinoma	18%	Accounts for 10% major and 33% of minor salivary gland malignant tumors
Malignant mixed tumor	13%	
Acinic cell carcinoma	7%	Accounts for 10% of malignant parotid gland tumors
Squamous cell carcinoma	4%	Occurs in 5-10% of parotid and submaxillary gland malignant tumors
Other	< 3%	

**Figure 3:**
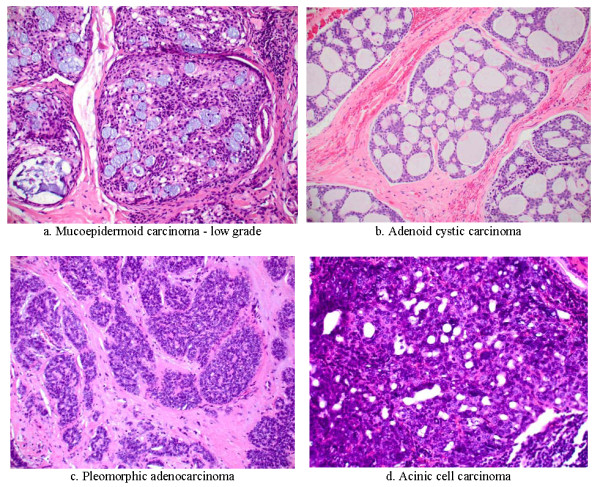
**Salivary gland malignant tumor pathology (200 ×)**.

The origin of parotid and other salivary gland cancers is still debated. Possibly, they are derived from both epithelial and mesenchymal elements, which may explain their variable patterns (See Figure 3a-d).

Most parotid gland cancers are slow-growing and treatable if found in the early stage. Prognosis varies according to histologic type and stage. A combination of radiation therapy and surgery is usually applied to treat this malignant tumor. Fatalities are usually not a direct result from the tumor, but typically occur due to metastasis to other organs, especially the lungs.

## Tumorigenesis Mechanism

The mechanism behind the development of parotid gland cancer is not fully understood. One theory involves vacuolar protein sorting-associated protein 4B homolog (VPS4B) [16]. This protein is responsible for vesicular trafficking and the maturation of autophagosomes in mammalian cells.

In humans, the epidermal growth factor receptor (EGFR) is a cell-surface receptor that is activated by binding of its specific ligands, including members of the epidermal growth factor (EGF) family of extracellular protein ligands [17,18]. Upon activation, EGFR transforms from an inactive monomeric form to an active homo/hetero-dimer. The dimerization stimulates its intrinsic intracellular protein-tyrosine kinase activity and results in auto-phosphorylation of several tyrosine residues in its C-terminal domain. This auto-phosphorylation elicits downstream activation and signaling to other proteins, which subsequently initiate several signal transduction cascades governing cell migration, adhesion, proliferation, differentiation, and death [19]. Controls of the intensity and duration of EGFR signaling are through a negative feedback regulatory mechanism of the EGF-induced EGFR downregulation process in which activated EGFR is endocytosed and then differentially destined for lysosomal degradation or recycling via the multi-vesicular bodies (MVBs) [20]. It is during this endocytic process that vacuolar protein sorting (VPS) associated-proteins play their role in EGFR regulation. In the formation of intracellular vesicles that bud into the MVBs, proteins are sorted into membrane micro domains, the membrane is distorted away from the cytoplasm, and the vesicle is released via membrane fission. MVB vesicle formation utilizes the cellular machinery of Class E VPS proteins [16]. There are 27 different human Class E proteins so far identified. Most of them function as subunits of the hetero-oligomeric endosomal sorting complexes required for transport (ESCRT) complexes, which are sequentially recruited to sites of vesicle formation. The major EGFR inactivation pathway of endocytosis utilizes four ESCRTs to culminate in lysosomal degradation of activated EGFR [21]. During endocytic process, VPS4, an ATPase associated with various cellular activities (AAA)-ATPase protein complex [22], is involved in the disassembly of the ESCRT-III complex, which is required for recycling of membrane-associated proteins in mammalian cells [23]. Two VPS4 isoforms, VPS4A and VPS4B, can hetero-oIigomerize with each other in mammalian cells. Endocytic pathway also converges with autophagic pathway, which is evolutionarily conserved and responsible for sequestering targeted proteins and organelles and their subsequent degradation in a lysosome-dependent manner [24]. While a special form of MVBs mediates the transport of cargo to lysosomes, recent studies demonstrated that the multisubunit complex, ESCRT-III, is required for autophagosomes to fuse with MVBs and for fusion of autophagosomes with lysosomes during autophagic process [25].

**Figure 4:**
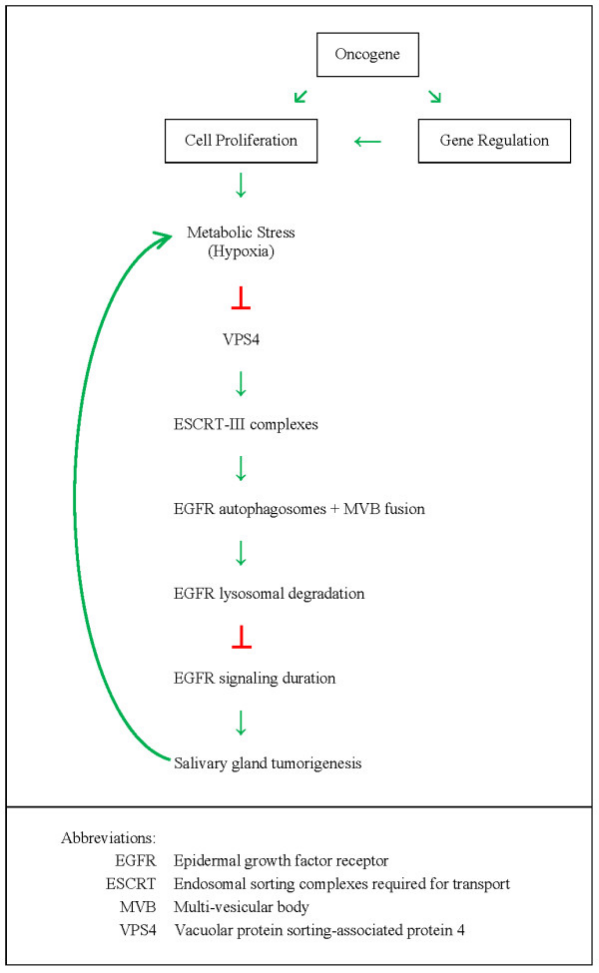
**Diagram of tumorigenesis mechanism**. Loss of VPS4 function promotes salivary gland tumorigenesis through its effect of prolonging the duration of EGFR signaling.

Given the essential role for VPS4B in MVB maturation, it is speculated that loss of VPS4 function might promote tumorigenesis through its effect of prolonging the duration of EGFR signaling (See Figure 4) [26].

Although increased levels of EGFR expression are observed in a variety of cancers, including head and neck, ovary, cervix, bladder, esophagus, stomach, brain, breast, colon, and lung, and frequently confer an adverse prognosis, but many cancers exhibited EGFR over-expression in the absence of EGFR gene amplification [27]. It is therefore postulated that the widespread phenomenon of EGFR over-expression in human cancers occurs, at least in part, as a consequence of common pathological events, other than genomic changes, associated with solid tumors.

## Treatment

Although most parotid gland tumors grow slowly and are noncancerous, they might continue to grow and can eventually become cancerous. Surgery with the complete removal the parotid gland (parotidectomy) and of the tumor, including a cuff of histologically normal tissue for adequate margins, is the mainstay treatment for parotid gland tumors. When there is direct extension of the primary tumor into the neck, neck dissection is necessary. The surgery can be complicated because several important nerves are located in and around the gland including the facial nerve (VII), which controls most facial movements. Malignant deep lobe parotid gland tumors require postoperative radiotherapy (RT) owing to the limitations of surgical margins in their resection. RT and chemotherapy/RT can also be considered as an adjuvant setting for tumors of intermediate or high grades. Studies [28,29] indicate that neutron-beam radiation is more effective than conventional radiation against malignant salivary gland disorders since it results in a higher degree of tumor destruction with fewer toxic effects to surrounding normal tissues. In treating advanced, recurrent or incompletely resected adenoid cystic carcinoma of the major and minor salivary glands, neutron-beam therapy [30] can achieve higher local control rate than photon therapy, and photon/neutron mixed beam. Thus, it is a recommended treatment to unresectable or inoperable cases. However, despite its better local control, neutron-beam radiation does not provide significant advantage in freedom from distant failure over photons or mixed beam radiotherapy. Furthermore, it does not have significant impact on survival, which is dominated by frequent distant metastases among advanced cases.

Chemotherapy is usually for palliation or confined to advanced disease. Responses have been reported after single-agent chemotherapy and polychemotherapy. Various agents, such as cisplatin, paclitaxel, cyclo-phosphamide, doxorubicin, mitoxantrone, carboplatin, and vinorelbine, and combinations of these have been shown to be active for some malignant histologies [31]. Table 2 indicates the results of various palliative mono- and poly-chemotherapy regimens for advanced cases [32-38].

**Table 2:** Results of palliative mono- and poly-chemotherapy to advanced salivary gland cancers

	**VNB **[32]	VNB/Cisplatin	**Cisplatin **[33]	**Paclitaxel **[34]	**Mitoxantrone/Cisplatin **[35]	**Cyclo-phosphamide/Doxorubicin/Cisplatin CAP **[36]	**Adriamycin/cis-Platinum/Cyclo-phosphamide **[37]	**Docetaxel **[38]
CR	0	3(19%)	3(12%)	0(0%)	0(0%)	3(23%)	3(60%)	2(50%)
PR	4(20%)	4(25%)	1(4%)	8(17·8%)	2(14·3%)	3(23%)	2(40%)	2(50%)
NC	9(45%)	6(37·5%)		15(33·3%)	9(64·3%)			
PD	7(35%)	3(19%)		20(44·4%)	3(21·4%)			
NE				2(4·4%)				
Patients	20	16	25	45	14	13	5	4

VNB: vinorelbine; CR: complete response; PR: partial response; NC: no change (stable disease); PD: progressive disease; NE: not evaluable

Although chemotherapy alone does not improve survival rates, the integration of radiation and chemotherapy has been shown to increase local control and represents an improvement in the management of parotid gland malignancies. Chemotherapy improves radiotherapy efficacy through its radio-sensitizing ability, and at the same time provides adjuvant systemic therapy against distant micro-metastasis [39]. Some of the concurrent chemotherapy and radiation therapy (chemoradiotherapy [CRT]) regimens have become standard treatments for locally advanced disease and high-risk pathology in definitive and postoperative setting, respectively.

Similar to those for other tumors, treatment selections for parotid gland tumor is largely dependent upon the extent and the pathology of the primary tumor as ascertained by clinical examination, pathology, and interpretation of appropriate radiographic images. According to the National Comprehensive Cancer Network (NCCN) 2011 Clinical Practice Guidelines in Oncology: Head and Neck Cancers [40], complete surgical excision is required for parotid gland carcinomas T1 &T2 [41], or clinically benign tumors, which are characterized by being mobile, superficial, slow growing, painless, with intact facial nerve (VII), and without neck nodes. No additional treatment is envisaged if the excised tumor is pathologically confirmed to be benign. However, if there is tumor spillage or the tumor is pathologically proven malignant, post-operation radiation therapy should be considered, regardless of grade.

For parotid gland carcinomas T3 and above, further surgical evaluation of local invasion or regional lymph node metastasis has to be performed. In the case of carcinoma T4b, it is either no surgical resection would be possible, or surgical resection is not recommended. Definitive radiation therapy or radiation therapy with systemic chemotherapy is recommended.

Among the clinical N_0 _(with no regional lymph node metastasis) tumors, parotidectomy with complete excision of tumor with or without neck dissection for high-grade and high-stage tumors is suggested, while the clinical N_+ _(positive regional lymph node metastasis) require parotidectomy and neck dissection. In the case of incomplete excision with gross residual disease during surgery, definitive radiation therapy or chemoradiation therapy is recommended. Even for complete excision, adjuvant radiation or chemoradiation therapy should also be considered.

## Future Developments

One of the reported side effects of radiation as treatment for parotid gland cancer is hearing loss. Clinical trials have been underway to determine whether intensity-modulated radiation therapy (IMRT) that spares the cochlear is more effective than conventional three-dimensional conformal radiation therapy (3D-CRT) in reducing sensory-neural hearing loss in patients who have radiotherapy treatment in the parotid gland region. Data are still being collected before definitive clinical guidelines can be put in place. Compared with IMRT and 3D-CRT, the new radiation treatment method of intensity-modulated proton therapy (IMPT) allows for optimal dose distribution to the local target, resulting in a dose reduction and improved sparing of the organs at risk, while keeping similar target coverage results [42]. Its potential benefit of lessening complications, such as salivary dysfunction, radionecrosis, or xerostomia, requires further clinical validation.

Thanks to the advances in molecular biology and our better understanding of the molecular mechanisms underlying head and neck cancers, including parotid gland cancer, new targeted therapeutics and novel agents are also being developed for the systemic treatment to them. The epidermal growth factor receptor (EGFR) is one of the major targets under intensive investigation since it has been found to be overexpressed in head and neck squamous cell carcinoma. This overexpression has been linked to disease recurrence in which EGFR-dependent signaling pathways are activated, leading to tumor cell proliferation and anti-apoptosis. EGFR blockade has hence been proposed to inhibit tumor growth. Cetuximab [43], an immunoglobulin-G1 antibody against EGFR, and erlotinib, a small-molecule inhibitor of the intracellular tyrosine kinase domain of EGFR, are among the novel targeted therapeutic agents to result in improved survival in patients with head and neck squamous cell carcinoma. Furthermore, to target the vascular endothelial growth factors (VEGF) and its receptors, which are vital to the angiogenesis for tumor growths, bevacizumab [44] and vanitinib, a monoclonal antibody against VEGF and VEGF receptor-2 inhibitor, respectively, are being clinically evaluated.

## Conclusion

Although parotid gland cancer is relatively rare, it still constitutes as a serious health hazard burdening our population because of its poor prognosis. Twenty percent of all patients will develop distant metastases despite its large variety of histologic types. Distant metastases usually correspond to a poor prognosis with a median survival of 4 · 3-7 · 3 months [45]. Patients with high-grade tumors have a higher chance of developing distant metastases than those with lower-grade tumors. Nonetheless, with more being unraveled about the disease, including its progression mechanisms at molecular level and novel treatment regimens being developed, we remain optimistic that locoregional control and overall patient survival will improve in not too distant future.

## List of abbreviations

3D-CRT: Three-dimensional conformal radiation therapy; ATPase: Adenosine triphosphate enzyme; CRT: Chemoradiotherapy; EGF: Epidermal growth factor; EGFR: Epidermal growth factor receptor; ESCRT: Endosomal sorting complexes required for transport; IMPT: Intensity-modulated proton therapy; IMRT: Intensity-modulated radiation therapy; MVB: Multi-vesicular body; RT: Radiotherapy; VEGF: Vascular endothelial growth factor; VPS: Vacuolar protein sorting; VPS4B: Vacuolar protein sorting-associated protein 4B.

## Competing interests

The authors declare that they have no competing interests.

## Authors' contributions

KH did literature review and wrote the paper. HL and DA devised the proposed tumorigenesis mechanism. PC provided the pathology images. YY conceptualized and designed the paper. All authors read and approved the final manuscript.

## References

[B1] Gray H Anatomy of the Human Body Splanchnology, Section 2a. The Mouth 2000 Chapter 11 Philadelphia: Lea & Febiger, 1918; Bartleby.com http://www.bartleby.com/107/illus1024.html Last accessed 26^th ^July 2011

[B2] Horner MJ Ries LAG Krapcho M (eds) SEER Cancer Statistics Review, 1975-2006, National Cancer Institute. Bethesda, MD 2009 http://seer.cancer.gov/csr/1975_2006/index.html Last accessed 26^th ^July 2011

[B3] Spitz MR Sider JG Newell GR Batsakis JG Incidence of salivary gland cancer in the United States relative to ultraviolet radiation exposure Head Neck Surg 1988 10 205 308 10.1002/hed.2890100504 3220771

[B4] Surveillance, Epidemiology, and End Results (SEER) Program http://www.seer.cancer.gov SEER*Stat Database: Incidence - SEER 17 Regs Research Data + Hurricane Katrina Impacted Louisiana Cases, Nov 2010 Sub (2000-2008), National Cancer Institute, DCCPS, Surveillance Research Program, Cancer Statistics Branch, released April 2011, based on the November 2010 submission http://seer.cancer.gov/canques/incidence.html, http://canques.seer.cancer.gov/cgi-bin/cq_submit?dir=seer2008&db=6&rpt=TAB&sel=^0^4^0^0^^0&y=Sex^1,2&z=Statistic%20type^1,2,3&dec=2,2,2&template=null Last accessed 26^th ^July 2011

[B5] Salivary Gland Cancer Detailed Guide. Learn About Cancer from the American Cancer Society http://www.cancer.org/Cancer/SalivaryGlandCancer/DetailedGuide/salivary-gland-cancer-what-is-key-statistics Last accessed 26^th ^July 2011

[B6] Rubin R Strayer DS (eds) Rubin's Pathology: Clinicopathologic Foundations of Medicine 2004 4 Lippincott Williams & Wilkins, Maryland 1283 8521922616

[B7] Davidson TM Clinical Manual of Otolaryngology 1992 3 University of California Medical School, San Diego, California, Copyright 2006 Regents University of California. Formerly published under the title Clinical Manual of Otolaryngology, McGraw-Hill Inc http://drdavidson.ucsd.edu/Portals/0/CMO/CMO_index.htm Last accessed 26^th ^July 2011

[B8] Salivary Gland Cancer Overview. Learn About Cancer from the American Cancer Society http://www.cancer.org/Cancer/SalivaryGlandCancer/OverviewGuide/salivary-gland-cancer-overview-survival-rates Last accessed 26^th ^July 2011

[B9] Saku T Hayashi Y Takahara O Salivary gland tumors among atomic bomb survivors, 1950-1987 Cancer 1997 79 1465 75 10.1002/(SICI)1097-0142(19970415)79:8<1465::AID-CNCR4>3.0.CO;2-A 9118025

[B10] Spitz MR Tilley BC Batsakis JG Gibeau JM Newell GR Risk factors for major salivary gland carcinoma: a case-comparison study Cancer 1984 54 1854 1859 10.1002/1097-0142(19841101)54:9<1854::AID-CNCR2820540915>3.0.CO;2-1 6478421

[B11] Schneider AB Favus MJ Stachura ME Salivary gland neoplasms as a late consequence of head and neck irradiation Ann Intern Med 1977 87 160 164 889197 10.7326/0003-4819-87-2-160

[B12] Horn-Ross PL Ljung BM Morrow M Environmental factors and the risk of salivary gland cancer Epidemiology 1997 8 4 414 419 10.1097/00001648-199707000-00011 9209856

[B13] Mancuso TF Brennan MJ Epidemiological considerations of cancer of the gallbladder, bile ducts and salivary glands in the rubber industry Journal of Occup Med 1970 12 333 341 5482047

[B14] McKenna RJ Tumors of the major and minor salivary glands CA Cancer J Clin 1984 34 1 24 39 10.3322/canjclin.34.1.24 6420017

[B15] Lalwani Anil K Malignant Diseases of the Salivary Glands Current diagnosis & treatment in otolaryngology: head & neck surgery 2008 Chapter 18 McGraw-Hill Medical21922533

[B16] Scott A Chung H Gonciarz-Swiatek M Structural and mechanistic studies of VPS4 proteins The European Molecular Biology Organization Journal 2005 24 20 3658 3668 10.1038/sj.emboj.7600818 PMC1276703 16193069

[B17] Schlessinger J Cell signaling by receptor tyrosine kinases Cell 2000 103 211 225 10.1016/S0092-8674(00)00114-8 11057895

[B18] Simon MA Receptor tyrosine kinases: specific outcomes from general signals Cell 2000 103 13 15 10.1016/S0092-8674(00)00100-8 11051543

[B19] Li E Hristova K Role of receptor tyrosine kinase transmembrane domains in cell signaling and human pathologies Biochemistry 45 20 6241 51 10.1021/bi060609y PMC4301406 16700535

[B20] Sorkin A Von Zastrow M Signal transduction and endocytosis: close encounters of many kinds Nut Rev Mol Cell Biol 2002 3 600 614 10.1038/nrm883 12154371

[B21] Katzmann DJ Odorizzi G Emr SD Receptor downregulation and multivesicular-body sorting Nut Rev Mol Cell Biol 2002 3 893 905 10.1038/nrm973 12461556

[B22] Esclatine A Chaumorcel M Codogno P Macroautophagy signaling and regulation Curr Top Microbiol Immunol 2009 335 33 70 10.1007/978-3-642-00302-8_2 19802559

[B23] White SR Lauring B AAA+ ATPases: achieving diversity of function with conserved machinery Traffic 2007 8 1657 1667 10.1111/j.1600-0854.2007.00642.x 17897320

[B24] Klionsky DJ Emr SD Autophagy as a regulated pathway of cellular degradation Science 2000 290 1717 1721 11099404 10.1126/science.290.5497.1717 PMC2732363

[B25] Rusten TE Vaccari T Lindmo K ESCRTs and Fabl regulate distinct steps of autophagy Curr Biol 2007 17 1817 1825 10.1016/j.cub.2007.09.032 17935992

[B26] Chen N Debnath J Autophagy and tumorigenesis Federation of European Biochemical Societies Letters 2010 584 1427 1435 10.1016/j.febslet.2009.12.034 20035753 PMC2843775

[B27] Santarius T Shipley J Brewer D A census of amplified and overexpressed human cancer genes Nut Rev Cancer 2010 10 59 64 10.1038/nrc2771 20029424

[B28] Laramore GE Krall JM Griffin TW Neutron versus photon irradiation for unresectable salivary gland tumors: Final report of an RTOG-MRC randomized clinical trial. Radiation Therapy Oncology Group. Medical Research Council Int J Radiat Oncol Biol Phys 1993 27 2 235 240 10.1016/0360-3016(93)90233-L 8407397

[B29] Stelzer KJ Laramore GE Griffin TW Fast neutron radiotherapy The University of Washington experience. Acta Oncol 1994 33 3 275 80 10.3109/02841869409098417 8018355

[B30] Huber PE Debus J Latz D Radiotherapy for advanced adenoid cystic carcinoma: neutrons, photons or mixed beam? Radiother Oncol 2001 59 2 161 7 10.1016/S0167-8140(00)00273-5 11325445

[B31] Laurie SA Licitra L Systemic therapy in the palliative management of advanced salivary gland cancers J Clin Oncol 2006 24 17 2673 8 10.1200/JCO.2005.05.3025 16763282

[B32] Airoldi M Pedani F Succo G Phase II randomized trial comparing vinorelbine versus vinorelbine plus cisplatin in patients with recurrent salivary gland malignancies Cancer 2001 91 3 541 7 10.1002/1097-0142(20010201)91:3<541::AID-CNCR1032>3.0.CO;2-Y 11169936

[B33] Licitra L Marchini S Spinazzè S Cisplatin in advanced salivary gland carcinoma. A phase II study of 25 patients Cancer 1991 68 1874 1877 10.1002/1097-0142(19911101)68:9<1874::AID-CNCR2820680904>3.0.CO;2-S 1913539

[B34] Gilbert J Li Y Pinto HA Jennings T Phase II trial of taxol in salivary gland malignancies (E1394): a trial of the Eastern Cooperative Oncology Group Head Neck 2006 28 3 197 204 10.1002/hed.20327 16470745

[B35] Gedlicka C Schüll B Formanek M Mitoxantrone and cisplatin in recurrent and/or metastatic salivary gland malignancies Anticancer Drugs 2002 13 5 491 5 10.1097/00001813-200206000-00007 12045460

[B36] Dreyfuss AI Clark JR Fallon BG Posner MR Norris CM Jr Miller D Cyclophosphamide, doxorubicin, and cisplatin combination chemotherapy for advanced carcinomas of salivary gland origin Cancer 1987 60 2869 2872 10.1002/1097-0142(19871215)60:12<2869::AID-CNCR2820601203>3.0.CO;2-Y 2824016

[B37] Alberts DS Manning MR Coulthard SW Koopmann CF Jr Herman TS Adriamycin/cis-platinum/Cyclophosphamide Combination Chemotherapy for Advanced Carcinoma of the Parotid Gland Cancer 1981 47 4 645 8 10.1002/1097-0142(19810215)47:4<645::AID-CNCR2820470404>3.0.CO;2-A 7194729

[B38] Raguse JD Gath HJ Bier J Riess H Oettle H Docetaxel (Taxotere) in recurrent high grade mucoepidermoid carcinoma of the major salivary glands Oral Oncol 2004 40 2 5 7

[B39] Brizel DM Esclamado R Concurrent chemoradiotherapy for locally advanced nonmetastatic, squamous carcinoma of the head and neck: consensus, controversy, and conundrum J Clin Oncol 2006 24 2612 2617 10.1200/JCO.2005.05.2829 16763273

[B40] NCCN Clinical Practice Guidelines in Oncology (NCCN Guidelines™) Head and Neck Cancers. Version 1.2011. NCCN.org 2011 National Comprehensive Cancer Network. lnc http://www.nccn.org/professionals/physician_gls/pdf/head-and-neck.pdf Last accessed 26th July 2011 10.6004/jnccn.2020.0031 32634781

[B41] Edge SB Byrd DR Compton CC Fritz AG Greene FL Trotti A (eds) American Joint Committee on Cancer (AJCC): AJCC Cancer Staging Manual 2010 7 New York, Berlin, Heidelberg: Springer-Verlag

[B42] van de Water TA Lomax AJ Bijl HP Potential benefits of scanned intensity-modulated proton therapy versus advanced photon therapy with regard to sparing of the salivary glands in oropharyngeal cancer Int J Radiat Oncol Biol Phys 2011 79 4 1216 24 Epub 2010 10.1016/j.ijrobp.2010.05.012 20732761

[B43] Bonner JA Harari PM Giralt J Radiotherapy plus cetuximab for locoregionally advanced head and neck cancer: 5-year survival data from a phase 3 randomized trial, and relation between cetuximab-induced rash and survival Lancet Oncol 2010 11 1 21 8 Epub 2009 10.1016/S1470-2045(09)70311-0 19897418

[B44] Cohen EE Davis DW Karrison TG A phase II study erlotinib and bevacizumab in patients with recurrent or metastatic squamous-cell carcinoma of the head and neck Lancet Oncol 2009 10 3 247 57 10.1016/S1470-2045(09)70002-6 19201650 PMC2768532

[B45] Schwentner I Obrist P Thumfart W Sprinzl G Distant metastasis of parotid gland tumors Acta Otolaryngol 2006 126 4 340 5 10.1080/00016480500401035 16608783

